# Development and validation of an intuitive biomechanics-based method for intraocular pressure measurement: a modal analysis approach

**DOI:** 10.1186/s12886-023-02867-8

**Published:** 2023-03-27

**Authors:** Francis Li-Tien Hsu, Po-Jen Shih, I.-Jong Wang

**Affiliations:** 1 College of Medicine, National Taiwan University, 10048 Taipei, Taiwan; 2 Department of Biomedical Engineering, National Taiwan University, 10048 Taipei, Taiwan; 3 Department of Ophthalmology, National Taiwan University Hospital, 10048 Taipei, Taiwan

**Keywords:** Biomechanics, Modal analysis, Cornea vibration

## Abstract

**Background:**

Current intraocular pressure (IOP) measurements based on non-contact tonometry are derived from statistics-driven equations and lack biomechanical significance, which often leads to under-estimation in post-refractive surgery cornea. This study aims to introduce and validate modal analysis-derived intraocular pressure (mIOP) as a novel method generated through Legendre-based modal decomposition of the anterior corneal contour; it provides an accurate and intuitive IOP measurement from an energy-based perspective.

**Methods:**

This retrospective study included 680 patients. Healthy participants were divided into reference (*n* = 385) and validation (*n* = 142) datasets, and the others underwent either femtosecond-assisted laser in situ keratomileusis (FS-LASIK, *n* = 58) or transepithelial photorefractive keratectomy (TPRK, *n* = 55). Corneal curvature of the right eyes was extracted from raw serial cross-sectional images of the cornea generated by Corvis ST, a noncontact tonometer with a high-speed Scheimpflug-camera. Legendre expansion was then applied to the corneal curvature to obtain the modal profiles (i.e., temporal changes of the coefficient for each basis polynomial [modes]). Using the reference dataset, feature selection on the modal profiles generated a final mIOP model consisting of a single parameter: total area under curve (frames 1–140) divided by the area under curve of the rising phase (frames 24–40) in the fourth mode, i.e. the M_4_ ratio. Validation was performed in both the healthy validation and postoperative datasets. IOP-Corvis, pachymetry-corrected IOP, biomechanically corrected IOP, and mIOP values were compared. For the FS-LASIK and TPRK groups, pairwise postoperative IOP changes were analyzed through repeated measures analysis of variance, and agreement was examined through Bland–Altman analysis. Using a finite element analysis based three-dimensional model of the human cornea, we further compared the M_4_ ratio with the true intraocular pressure within the physiological range.

**Results:**

The M_4_ ratio-based mIOP demonstrated weak to negligible association with age, radius of corneal curvature, and central corneal thickness (CCT) in all validation analyses, and performed comparably with biomechanically corrected IOP (bIOP) in the refractive surgery groups. Both remained nearly constant postoperatively and were not influenced by CCT changes. Additionally, M_4_ ratio accurately represented true intraocular pressure in the in silico model.

**Conclusions:**

mIOP is a reliable IOP measurement in healthy and postrefractive surgery populations. This energy-based, ratio-derived approach effectively filters out pathological, rotational, misaligned movements and serves as an interpatient self-calibration index. Modal analysis of corneal deformation dynamics provides novel insights into regional corneal responses against pressure loading.

**Supplementary Information:**

The online version contains supplementary material available at 10.1186/s12886-023-02867-8.

## Background

Elevated intraocular pressure (IOP) is associated with various ophthalmological diseases, particularly with glaucoma [1]. First introduced in 1948, the Goldmann applanation tonometer (GAT) remain the gold standard for measuring IOP [2]. Based on the Imbert–Fick law, the GAT analyzes the static equilibrium between applanation force and the IOP through application of a truncated cone, which flattens a circular area on the corneal surface at a force directly proportional to IOP. However, in recent years, the GAT has largely been replaced by noncontact tonometry, which combines delivery of a puff of air that briefly deforms the cornea and an optical electrical device that quantifies the deformation. The Ocular Response Analyzer, a noncontact tonometry device, offers two IOP measurements: Goldmann-correlated IOP and corneal-compensated IOP, the latter based on corneal hysteresis correction [3]. Another noncontact tonometry, Corvis ST (OCULUS, Germany), measures IOP based on electro-optical system–assisted observation of corneal applanation during dynamic equilibrium, namely using an ultrahigh speed Scheimpflug camera to capture serial cross-sectional images of corneal deformation throughout the air-puff. Additionally, it reports the time, length, and velocity of the first and second applanation moments, along with several morphological parameters describing corneal curvature at the highest concavity, including deformation amplitude, curvature radius, and peak distance. These parameters are reflective of in vivo corneal biomechanics, and have been widely applied in studying corneal diseases and the material properties of the human cornea [4]. However, such IOP measurements are typically overestimated due to the momentum of the deforming cornea and the compression of the anterior chamber during air-puff loading [5, 6] and require further correction [7]. Accordingly, both static- and dynamic-based tonometry can be affected by various corneal parameters, including central corneal thickness (CCT), changes or irregularities in the corneal curvature, tear film status, aging, and refractive surgery [2].

To obtain accurate IOP measurements from noncontact tonometry, numerous correction methods for different devices have been developed, and their resulting values have been compared with those of the GAT. These correction equations are largely derived through either linear [8] or high-order nonlinear regression [9] and primarily focus on minimizing the influence of three factors: CCT, age, and radius [9–13], with CCT as the main target for correction given the lack of consensus on how IOP should be measured among patients who underwent refractive surgery [14–16]. One proposed solution, the CCT-adjusted IOP (IOP-Pach) is generated by the Corvis ST software using the following equation: [corrected IOP = measured IOP + k − age (550 − CCT)] [17]. Although theoretically it would reduce the effect of corneal biomechanical properties on IOP value, previous studies have suggested that it remain dependent on the biomechanical parameters [18]. Another method, the biomechanically corrected IOP (bIOP), is based on the regression of 750 patients and accounts for corneal thickness at three positions as measured by Corvis ST [10, 19, 20], as well as patients’ ages and corneal curvatures. The final bIOP equation consists of 19 coefficients [10]. Modifications of the bIOP for patients with soft corneas or keratoconus were also published [21]. The bIOP is independent of CCT but remain dependent on corneal hysteresis and corneal resistance factor [19].

In summary, corneal deformation from an external air puff in noncontact tonometry offers invaluable information on corneal biomechanical characteristics in vivo, yet past studies have mostly focused on limited properties derived from only a few specific timepoints (e.g., at the highest concavity or first applanation) [22]. Consequently, estimations of IOP from these studies have failed to fully reflect the dynamic time-varying equilibrium between the air puff and IOP. The resulting models, therefore, are either oversimplified and unapplicable to postrefractive surgery populations, as in the case of IOP-Corvis, or are based on exhaustive parameterized modeling with complex equations such that no clear physical meaning can be derived, as is the case with the bIOP.

One of the key factors affecting the values of IOP is the corneal thickness; a good IOP should not be affected by corneal thickness. To elucidate how IOP affects corneal biomechanics, we sought to identify temporal changes in cornea behavior under air-puff induced deformation by applying “modal analysis” (i.e., a Legendre polynomial, expansion-based decomposition model) to the anterior cornea profiles of healthy individuals and patients receiving refractive surgery. Even modes reflecting IOP-related corneal bending were included in the final model, whereas high-order and odd modes representing air-puff misalignment were filtered out.

## Methods

### Participants and measurements

This is a single site, retrospective observational study comprising 680 participants enrolled at Dr. Lin’s Eye Clinic (a local ophthalmology clinic in Taoyuan, Taiwan) between March 2012 and December 2019. The datasets are split into two: dataset A serves as the exploration dataset in which IOP-specific modal features are defined and selected to generate the mIOP model; dataset B serves as the validation dataset to examine the applicability of the model on a random population. We investigated the sequential cross-sectional Scheimpflug images of each eye. Several parameters, such as patient age, CCT, the time to the first applanation (A1T), the corneal curvature radius (R), and the IOP, were collected from medical records, if available. IOP measurements reported by Corvis ST were recorded, which include IOP-Corvis, CCT-adjusted IOP (IOP-Pach), and bIOP. All examinations were performed by the same experienced ophthalmologist and technicians.

All patients received a complete ophthalmological examination, including a refraction test, slit lamp examination, and Corvis ST dynamic Scheimpflug analyzer test upon enrollment, and their cornea deformation response were recorded. Among these patients, 385 healthy participants, who had enrolled before November 1, 2015, were selected as the reference dataset (Dataset A) for establishing our model for physiological IOP estimation. Another 142 healthy participants who enrolled after November 1st, 2015 were selected as the validation dataset (Dataset B). To mitigate the possibility of inter-eye asymmetry due to pathological conditions, the inclusion criteria for both eyes were IOP-Corvis between 8 and 30 mmHg, no previous ocular surgery or disease, myopia of less than 10.0 diopters, and a corneal curvature radius of less than 20 mm. Due to the high correlation of all parameters between healthy bilateral eyes, only measurements of the right eye were used for analysis. In total, 369 and 129 eyes were included in Datasets A and B, respectively.

A third independent dataset (Dataset C) comprised 113 patients who underwent either femtosecond-assisted laser in situ keratomileusis (FS-LASIK, *n* = 58) or transepithelial photorefractive keratectomy (TPRK, *n* = 55) for primary myopic or myopic refractive correction. The inclusion criteria were complete follow-up measurements and no previous ocular surgery, disease, chronic medication use, or irregular astigmatism. The preoperative and 1-month postoperative measurements for each patient’s eyes were compared and analyzed to evaluate the performance of our IOP model. Similarly, only the right eyes were selected for analysis, with 41 and 48 eyes being included in the FS-LASIK and TPRK groups, respectively.

### Data analysis and development of the modal analysis workflow

Raw images of each frame were preprocessed with edge detection and artifact removal via custom-written programs in MATLAB software (version 2019b; The MathWorks, Natick, MA, USA). Additional file 1 describes the image processing workflow (including segmentation, noise filtering, and binarization) in detail. Blurry images, distorted images, or measurements with low Corvis ST quality (modal deviation) were excluded from further analysis.

Curve fitting via smoothing spline function was applied to fit the anterior cornea profile in each of the 140 frames spanning approximately 32 ms from the Corvis ST measurement. Next, Legendre expansion was employed to decompose individual profiles into orthogonal and complete sets of basis polynomials. For decomposition of the deflection curves, we considered the time-domain variations of the anterior cornea profile as the product of the spatial and time functions. The solution of the anterior deflection curve $u\left(\theta , {t}_{i}\right)$ acquired from the image, is represented by.

1 $$u\left(\theta,t_i\right)={\textstyle\sum_{n=0}^\infty}\;P_n\left(\theta\right)a_n(t_i)$$

where modal order *n* is the number of nodes on the curves, $\theta$ is the angle, *t*_i_ is the *i*-th time step during the air puff, ${P}_{n}(\theta )$ is the Legendre polynomials. ${a}_{n}({t}_{i})$ coefficient represents modal displacement, which is obtained by

2 $${a}_{n}({t}_{i})=\frac{2n+1}{2}{\int }_{R}u\left(\theta , {t}_{i}\right){P}_{n}\left(\mathrm{cos}\theta \right)\mathrm{sin}\theta d\theta$$

Here *R* is the region of integral points on the corneal surface curve, roughly -0.6 ~ 0.6 rads depending on the images.

**Fig. 1:**
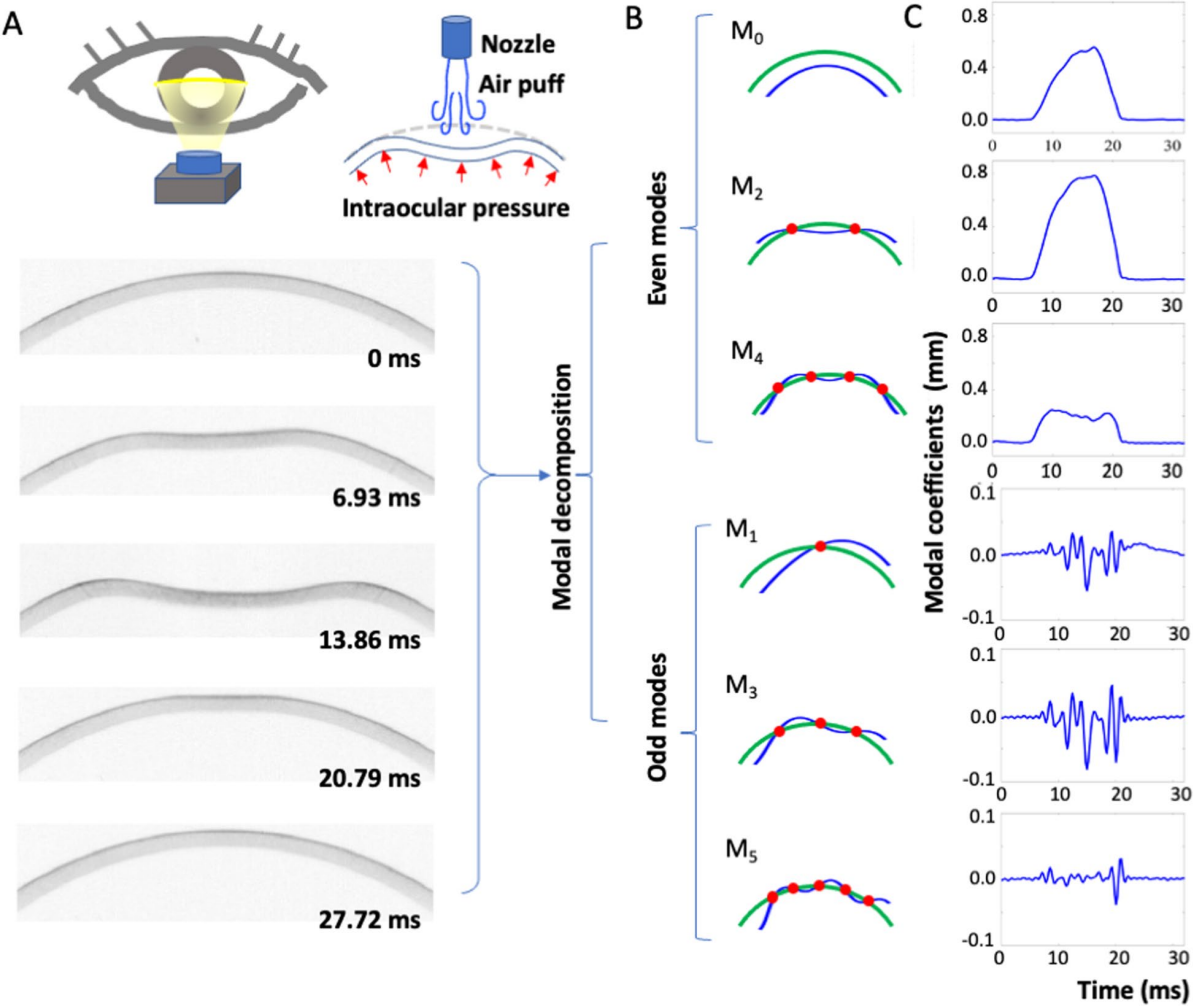
Schematic representation of the proposed Legendre-based modal analysis approach. (**A**) Cross-sectional images of corneal deformation. The outer corneal contour is decomposed into even and odd modes, their respective modal shapes as shown in (**B**). Modal profile (i.e., temporal changes of the coefficient for each basis polynomial [modes]) for each mode are shown in (**C**)

Based on the Akaike Information Criterion and empirical trial-and-error, all polynomial calculations of modal coefficients were limited up to the fifth degree to minimize unnecessary noise and overfitting. Legendre decomposition resulted in even (i.e., symmetrical modes M_0_, M_2_, and M_4_) and odd (i.e., antisymmetrical modes M_1_, M_3_, and M_5_) modes. The respective waveform for each mode is shown in Fig. 1B. Throughout the entire deformation, the temporal change of each modal coefficient is shown in Fig. 1C.

From the modal profiles, we observed that both the initial and final “resting” phases were relatively the same across all modes, whereas the cornea oscillation phase varied considerably. Feature selection, primarily in the form of classical waveform analysis, was then performed for each of the six modal curves. For each of the six modal profiles, we focused on selecting physical features that is descriptive of the time-varying coefficient change, such as the initial/final/maximal displacements, slope, area under curve, etc. We observed empirically on how modal profiles of different patients change with increasing IOP, and identified these parameters as representative of the phase changes and conserved “events” across different modes. A complete definition of all features included in our study is shown in Additional file 3. This approach was adopted to identify as many biomechanically significant signals as possible and to fully uncover how the relative weight of each mode alters during deformation and, in doing so, to physically interpret IOP through these parameters.

Given previous revelations that odd modes reflect pathological regional cornea instabilities, air-puff misalignment, or rotational deviations during measurement [23, 24] and M_0_ has been established as being the “breathing mode” (i.e., representing whole eye movement [WEM]), the symmetrical bending even modes M_2_ and M_4_ were therefore identified as being of particular interest (Fig. 1B). The contribution of the other high–order modes to overall corneal strength is negligible due to their modal shapes being too tortuous to reflect actual deformation and thus unlikely to play any role in the modal analysis–based IOP model. As the dynamic interaction among the external air puff, intrinsic corneal biomechanics, and intraocular pressure occurs in a symmetrical and balanced condition [25], we hypothesized that one or more parameters extracted from M_2_ and M_4_ could serve as suitable models for IOP estimation and offer straightforward physical insight into the relationship between corneal deformation and IOP (see Additional file 3 for a complete list of extracted parameters).

To identify potential candidates for construction of an IOP model derived from modal analysis, we performed simple linear regression and examined the degree of correlation between each parameter and the clinical variables (e.g., age, CCT, R, A1T, IOP-Corvis, and IOP-Pach) within the reference dataset. Ideal candidates who exhibited significantly high degrees of correlation with A1T and IOP but moderate to low correlation with the other variables were further selected. To avoid multicollinearity, the correlations between these candidates were analyzed. Suitable parameters were then included in the multivariate linear regression analysis to generate a final modal analysis–derived IOP (mIOP) model.

### Clinical validation

To assess the performance of the mIOP model as an effective indicator of true IOP, the same modal analysis procedure was repeated for two independent validation datasets (Datasets B and C), and mIOP values were obtained for each measurement. First, we quantitatively analyzed the population distribution of three IOP measurements (mIOP, IOP-Corvis, and IOP-Pach) in the healthy dataset (Dataset B). Next, mIOP was compared against IOP-Corvis and IOP-Pach with respect to the relationship with the independent variables of age, CCT, R, and A1T by using univariate linear regression, as evaluated using the Pearson correlation coefficient, estimated β value (slope), and p value. Finally, the extra sum-of-squares *F* test was employed to determine if a horizontal line would fit better as an alternative model (i.e., no significant association existed between the two variables).

For both the FS-LASIK and TPRK patients in Dataset C, the changes in the pairwise pre- and postoperative measurements of the IOP-Corvis, IOP-Pach, bIOP, and mIOP and their resulting distributions were analyzed using repeated measures analysis of variance (ANOVA) to investigate whether any method outperformed the others in terms of minimizing pre- and post-operative IOP differences. This is based on the assumption that in the absence of other ocular pathologies, the real IOP (i.e., the pressure exerted by the aqueous humor of the anterior chamber on the cornea inner surface) should theoretically remain constant throughout. The relationship between ΔIOP and ΔCCT was examined using univariate linear regression to determine the effect of corneal thinning on IOP measurements. As with Dataset B, univariate linear regression was performed on the clinical variables and the three IOP values, followed by an extra sum-of-squares *F* test. Finally, Bland–Altman analysis was applied to Datasets B and C to evaluate the relative agreement between parallel IOP methods.

### In silico validation

An in silico three-dimensional model of the dynamic corneal deformation process was simulated using COMSOL Multiphysics software. To do so, we applied a time-varying air puff field on a corneal model whose morpho-geometric parameters are comparable with real-world normal human cornea [26]. A detailed account of this model has been documented in our previous work [27]. For material-related parameters, both the hyperelastic and viscioelastic corneal characteristics were taken into consideration. The air puff velocity distribution is simulated by combining two logistical equations. Apart from time and spatial dependence, a delay effect was also incorporated into the air velocity distribution. The air velocity profile is delayed on the surface regions that exceeds 0.4 mm away from the apex, and the delayed time is proportional to the distance from the apex. For a complete list of input parameter and their values, please refer to Additional file 2.

Through defining the mesh grid and computing the finite element analysis and fluid–solid interaction processes, the cross-sectional corneal deformation curve along the horizontal meridian was obtained. Within the normal intraocular pressure range (13-21 mmHg), M_4_ ratio derived from modal analysis was compared against true intraocular pressure.

### Statistical analysis

*P* value for baseline characteristics were based on independent t-test following Levene's test for homogeneity of variance. Univariate linear regression was used to assess correlation of modal–derived parameters in dataset A, as well as examining the intercorrelation of IOP measurements and corneal biomechanical variables in dataset B and C, and also the relationship between M_4_ ratio and true IOP in the in silico model.

*P* value based on extra sum-of-squares F test was employed to determine if a horizontal line would fit better as an alternative model. Paired IOP measurements were analyzed via repeated measures one-way ANOVA with Geisser-Greenhouse correction and post hoc Tukey’s test in datasets B and C. Bland–Altman analysis was employed to analyze agreement among IOP measurement methods in datasets B and C. All statistical analyses were performed using GraphPad Prism (version 6.04; GraphPad Software, La Jolla, CA, USA) and the Statistical Package for the Social Sciences (version 25.0; SPSS, Chicago, IL, USA). Two-tailed *p* values of less than 0.05 were considered statistically significant.

## Results

### Parameter selection and establishing the mIOP model

**Fig. 2:**
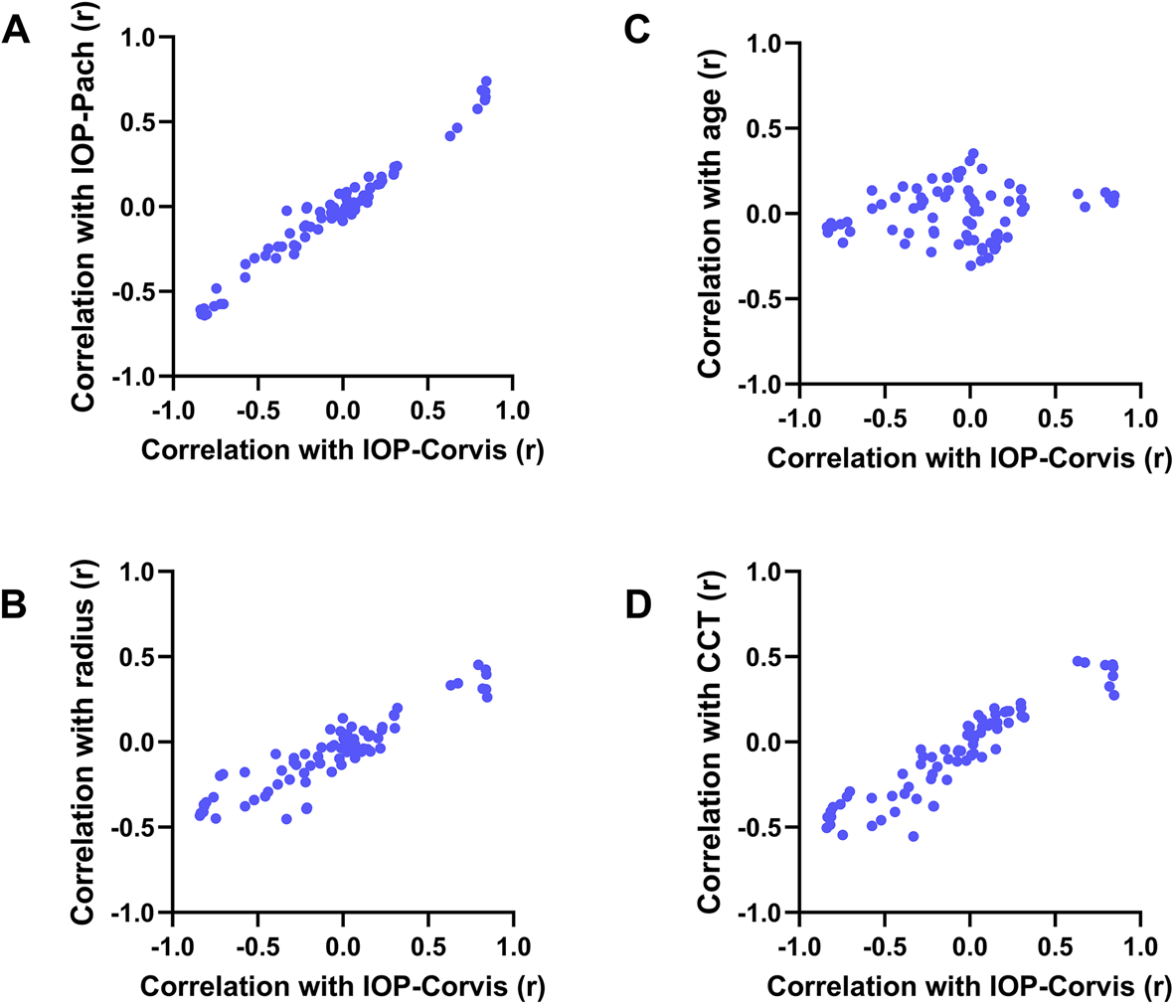
Relationship between degree of correlation with IOP-Corvis and degree of correlation with other corneal biomechanical variables for all 65 primary modal–derived parameters in the healthy reference dataset. Evaluated by Pearson’s correlation coefficient (r), scatter plots against IOP-Pach (**A**), radius (**B**), age (**C**), and CCT (**D**) are shown

A total of 65 modal parameters were acquired through the aforementioned process (complete definitions presented in Additional file 3). Univariate linear regression of each parameter with age, CCT, R, A1T, IOP-Corvis, and IOP-Pach revealed that the degree of correlation with both IOP measurements was positively associated with the correlation to CCT or R and, to a lesser but significant degree, with the correlation to age (Fig. 2). This suggests that the majority of modal parameters that correlate well with IOP actually reflect spurious correlation due to confounding variables, such as CCT and age, and thus remain highly dependent on the biomechanical properties of the cornea rather than being true IOP indicators in themselves.

We know that an accurate measurement of IOP must be independent of the geometric and stiffness characteristics of the cornea. Therefore, we sought to develop secondary parameters (based on combining and mathematically transforming the aforementioned primary parameters) to minimize the effect of CCT, age and corneal radius on IOP measurement. This was predominantly achieved through generating ratios by dividing primary parameters of the same physical quantity to extract potentially useful orthogonal properties. This also served as a self-calibration process that accounted for individual variations in deformation responses resulting from corneal properties, thereby allowing the standardization of such a comparison to be based solely on IOP differences. Moreover, because the temporal profile of the air puff remained constant in every test, the area under curve for each modal profile essentially represents the product of the external loading force and the modal displacement (i.e., the work done by the air puff in a given time interval). With this energy-based focus, we further observed the rising and downward phases of the modal profiles, since the relative contribution of even modes to the total deformation changes most significantly during these two phases. Rising phase refers to the time range from the start of air-puff induced deformation to first applanation, while downward phase refers to the time range from the second applanation to the end of air-puff induced deformation. Theoretically, these phases would reflect the difficulty in bending the corneal contour, which is itself a combination of material stiffness and IOP [28]. As depicted in Fig. 1B, two nodes separate the M_2_ modal shape into three segments, whereas three nodes separate M_4_ into five segments. The air-puff vector is most perpendicular to the corneal surface at the central one-third and one-fifth segments for M_2_ and M_4_, respectively. These are the major bended areas during air-puff deformation; on the other hand, deformation of the peripheral segments contributes less to the coefficients ${a}_{n}({t}_{i})$. Therefore, the major determinant of the n-th modal profile is deformation of the central one-(n + 1)th segment of the corneal contour, and are thus regions of great interest as the IOP and external loading falls on the same axis, allowing for a simplified force diagram as shown in Fig. 1A. The full mathematical forms of M_2_ and M_4_ are as follows:

3 $${a}_{2}({t}_{i})=\frac{5}{2}{\int }_{R}u\left(\theta , {t}_{i}\right){P}_{2}\left(\mathrm{cos}\theta \right)\mathrm{sin}\theta d\theta$$

4 $${a}_{4}({t}_{i})=\frac{9}{2}{\int }_{R}u\left(\theta , {t}_{i}\right){P}_{4}\left(\mathrm{cos}\theta \right)\mathrm{sin}\theta d\theta$$

Based on Additional file 3, we determined that the most suitable performance parameters are P30 and P32; these parameters have a comparatively weaker correlation with CCT, age and corneal radius. Among the secondary parameters listed in Table 1, a performance score was used to determine the potential candidate to construct a modal analysis-based IOP. Performance is mathematically defined as:

**Table 1:** All 10 secondary parameters with definitions and degrees of correlation with IOP-Corvis, IOP-Pach, age, R, CCT, and A1T in the healthy reference dataset

	$\frac{{AUC}_{\#1-\#140 Frames}}{{AUC}_{\#24-\#40 Frames}}$					$\frac{{AUC}_{\#1-\#140 Frames}}{{AUC}_{\#2nd applanation-\#140 Frames}}$				
Pearson’s r	M_1_	M_2_	M_3_	M_4_	M_5_	M_1_	M_2_	M_3_	M_4_	M_5_
IOP-Corvis	0.0460	0.8184	0.0147	0.8460	-0.0426	-0.0923	0.3575	0.0514	0.6062	-0.0118
IOP-Pach	0.0501	0.6864	-0.0254	0.7389	-0.0152	-0.0657	0.3488	0.0153	0.5932	-0.0089
Age	0.0593	0.0857	0.0723	0.1052	-0.0400	-0.0520	0.1456	0.0535	0.1622	-0.0123
R	0.0065	0.3123	0.0783	0.2617	0.0141	-0.0022	0.0272	0.0750	0.1408	-0.0441
CCT	0.0244	0.3261	0.0662	0.2740	-0.0460	-0.0436	0.0556	0.0337	0.0863	-0.0032
A1T	0.0459	0.8222	0.0101	0.8408	-0.0459	-0.0787	0.3621	0.0653	0.6074	-0.0055
**Performance**	0.0179	0.511	-0.077	0.578	-0.004	-0.0464	0.277	-0.0207	0.469	0.0095

*R* Corneal curvature radius, *CCT* Central corneal thickness, *A1T* First applanation time, *AUC* Area under curve

5 $$\mathrm{Performance}=\frac{1}{2}\left({r}_{IOP-Corvis}+{r}_{IOP-Pach}\right)-\frac{1}{3}({r}_{age}+{r}_{radius}+{r}_{CCT})$$

Theoretically, the most ideal parameter would result in a performance score of 1. Out of the 10 parameters, two were identified as the best performing: the ratio of the total modal curve area divided by the rising phase area in M_2_ and that in M_4_. The total area under the modal curve (AUC) is denoted by AUC_#1-#140 Frames_. The rising phase AUC is measured between the 24^th^ and 40^th^ frames, and is denoted by AUC_#24-#40 Frames_. The downward phase refers to the time elapsed from the second applanation to the end of air-puff induced deformation, and is denoted by AUC_#2nd applanation-#140 Frames_. These two are highly inter-correlated, likely due to overlaps in the observed regions (as previously discussed). Therefore, only the M_4_ ratio was used to generate the final mIOP model through simple linear regression, as presented in the following:

6 $$\mathrm{mIOP}=\left(0.586\times \frac{{\mathrm{M}}_{4} {AUC}_{\#1-\#140 Frames}}{{\mathrm{M}}_{4} {AUC}_{\#24-\#40 Frames}}\right)+ 8.2698\,(\mathrm{mmHg})$$

**Fig. 3:**
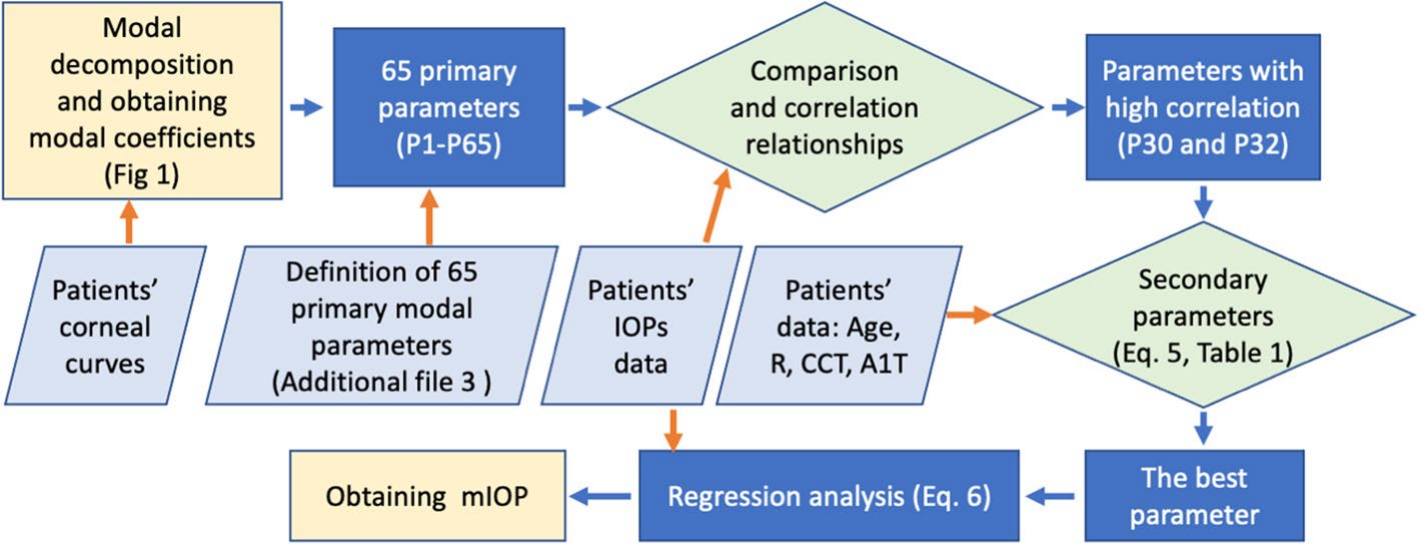
The flow chart of obtaining mIOP

A flowchart (Fig. 3) is provided to introduce the process.

### Validation in two independent datasets

The baseline characterization of Datasets A and B is as shown in Table 2. The average age and CCT were 39.39 ± 13.76 (years [mean ± S.D.]) and 543.41 ± 38.60 (µm), respectively, in the reference group (Dataset A) and 37.36 ± 14.14 (years) and 538.10 ± 38.19 (µm), respectively, in the validation group (Dataset B). No significant difference in age, CCT, R, A1T, or any of the three IOP measurements was observed between the two datasets. For Dataset B, the mIOP method produced the lowest values and the narrowest standard deviation range (14.27 ± 1.98 mmHg [mean ± S.D.]), followed by IOP-Pach (14.74 ± 2.70 mmHg) and IOP-Corvis (15.19 ± 2.504 mmHg). Through repeated measures ANOVA, significant differences were found between the three IOP methods (all *p* < 0.0001). Tukey’s multiple comparisons test revealed that mIOP values were significantly lower than those of IOP-Corvis (*p* < 0.01) and IOP-Pach (*p* < 0.0001).

**Table 2:** Baseline characterization of the healthy datasets

	**Reference Set (*n* = 369)**	**Validation Set (*n* = 129)**	***P* value **^**a**^
Age [yrs]	39.39 ± 13.76	37.36 ± 14.14	0.154
R [mm]	7.25 ± 1.21	7.26 ± 1.04	0.929
CCT [µm]	543.41 ± 38.60	538.10 ± 38.19	0.178
A1T [ms]	7.35 ± 0.40	7.37 ± 0.36	0.744
IOP-Corvis [mmHg]	14.53 ± 3.41	14.74 ± 2.70	0.523
IOP-Pach [mmHg]	14.82 ± 3.25	15.19 ± 2.50	0.245
mIOP [mmHg]	14.53 ± 2.89	14.27 ± 1.98	0.343

^a^ *p* values were calculated using the independent *t* test

**Fig. 4:**
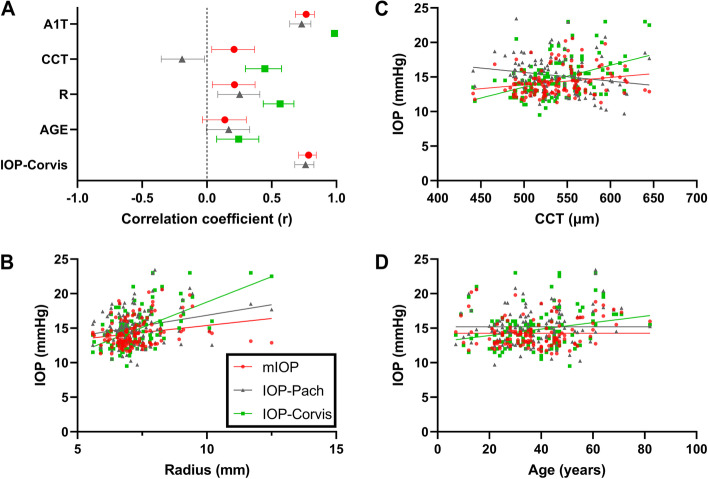
Comparison of three IOP methods (mIOP (red), IOP-Pach (black), and IOP-Corvis (red)) with respect to association with age, R, and CCT in the healthy validation dataset. Evaluated using univariate linear regression, (**A**) shows the Pearson’s correlation coefficient (r) with 95% confidence interval. The respective scatter plots and linear regression results with (**B**) radius, (**C**) CCT, and (**D**) age are also shown. Note that for (**D**), the linear regression line for IOP-Corvis and the horizontal fitting lines for mIOP and IOP-Pach are shown

Univariate linear regression (Table 3 & Fig. 4A) revealed that age was associated with IOP-Corvis (β = 0.04664, *r* = 0.2446, *p* = 0.0052) but not with IOP-Pach (β = 0.02972, r = 0.1678, *p* = 0.0573) or mIOP (β = 0.01942, *r* = 0.1384, *p* = 0.1179). For the IOP-Pach and mIOP, the horizontal line was better fitted, as displayed in Fig. 4B. Radius was associated with all three IOP methods, although mIOP arguably demonstrated a weaker association (β = 0.407, *r* = 0.2125, *p* = 0.0156) than either IOP-Pach (β = 0.6109, *r* = 0.2527, *p* = 0.0039) or IOP-Corvis (β = 1.47, *r* = 0.5649, *p* < 0.0001) did. The differences between the slopes of mIOP and IOP-Pach with respect to R were not significant (*p* = 0.4438), while their elevations differed significantly (*p* = 0.0009; Fig. 4C). Finally, CCT was also associated with the three methods (Fig. 4D), with IOP-Corvis demonstrating the strongest association (β = 0.03159, *r* = 0.4475, *p* < 0.0001) compared with mIOP (β = 0.01087, *r* = 0.2092, *p* = 0.0174) and IOP-Pach (β =  − 0.0126, *r* =  − 0.192, *p* = 0.0293); the slopes of mIOP and IOP-Pach with respect to CCT differed significantly (*p* = 0.0014).

**Table 3:** Univariate linear regression between the three IOP methods (mIOP, IOP-Corvis, and IOP-Pach) with age, R, CCT, and A1T, respectively, in the healthy validation dataset

	**mIOP**		**IOP-Corvis**		**IOP-Pach**	
	**β**	***p* value **^a, b^	**β**	***p* value **^a, b^	**β**	***p* value **^a, b^
**Age [yrs]**	0.01942	0.1179	0.04664	0.0052**	0.02972	0.0573
**R [mm]**	0.407	0.0156*	1.47	< 0.0001****	0.6109	0.0039**
**CCT [µm]**	0.01087	0.0174*	0.03159	< 0.0001****	-0.01259	0.0293*
**A1T [ms]**	4.263	< 0.0001****	7.44	< 0.0001****	5.134	< 0.0001****

^a^ *p* values were calculated using the F test

^b^ * *p* < 0.05, ** *p* < 0.01, *** *p* < 0.001, **** *p* < 0.0001

The baseline preoperative characteristics of Dataset C are as listed in Table 4. Significant differences were found in the R, CCT, and A1T between the FS-LASIK and TPRK groups. IOP-Corvis, bIOP, and mIOP values were all significantly higher in the FS-LASIK group (*p* = 0.003, = 0.043, < 0.0001, respectively). In the FS-LASIK group, repeated measures ANOVA indicated significant preoperative differences between the four IOP methods (*p* = 0.0006), Tukey’s multiple comparison test showed that the mIOP (14.03 ± 1.357 mmHg) value was significantly lower than IOP-Corvis and IOP-Pach and bIOP (*p* = 0.0082, 0.0005, 0.0006 respectively). Similar results were found for the TPRK group, with mIOP (12.86 ± 1.084 mmHg) being the lowest of all four IOP readings (all *p* < 0.0001).

**Table 4:** Baseline preoperative characterization of FS-LASIK and TPRK patients

	**FS-LASIK (*n* = 41)**	**TPRK (*n* = 48)**	***p* value **^a, b^
Age [yrs]	32.93 ± 9.22	34.52 ± 9.19	0.418
R [mm]	7.22 ± 0.94	6.75 ± 0.57	0.005**
CCT [µm]	534.41 ± 33.05	516.27 ± 26.49	0.005**
A1T [ms]	7.45 ± 0.23	7.17 ± 0.21	< 0.0001****
IOP-Corvis [mmHg]	14.65 ± 1.61	13.56 ± 1.68	0.003**
IOP-Pach [mmHg]	15.39 ± 2.03	15.60 ± 1.90	0.613
bIOP [mmHg]	14.70 ± 1.24	14.11 ± 1.47	0.043*
mIOP [mmHg]	14.03 ± 1.36	12.86 ± 1.08	< 0.0001****

^a^ *p* values were calculated using the independent *t* test

^b^* *p* < 0.05, ** *p* < 0.01, *** *p* < 0.001, **** *p* < 0.0001

**Fig. 5:**
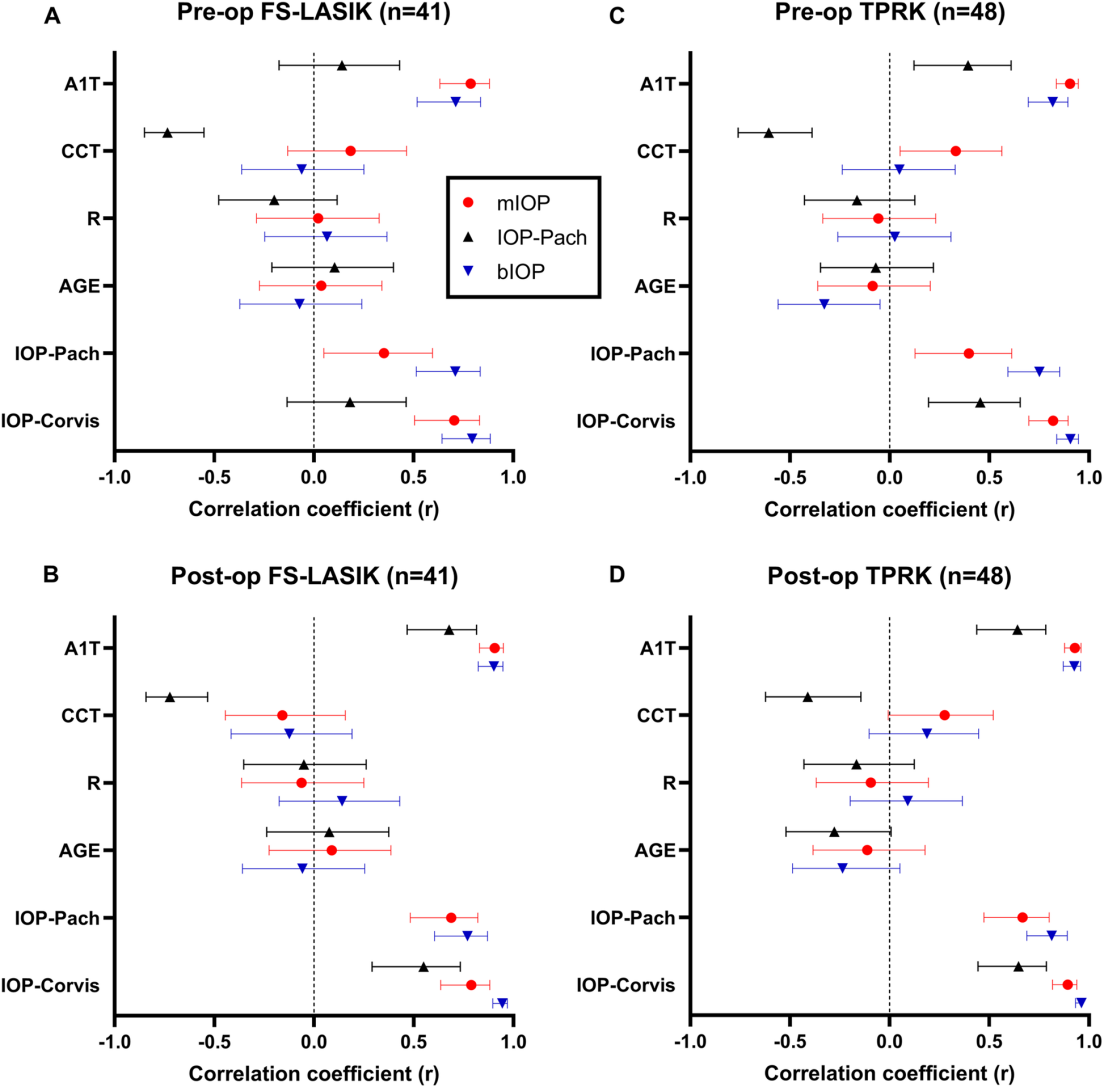
Comparison of three IOP methods (mIOP (red), IOP-Pach (black), and bIOP (blue)) with respect to association with A1T, age, R, and CCT in the refractive surgery dataset. Evaluated using univariate linear regression, Pearson’s correlation coefficient (r) with 95% confidence interval both preoperatively (**A**, **C**) and postoperatively (**B**, **D**) are shown

Univariate linear regression (Fig. 5 and Additional file 4) revealed that, pre-operatively, CCT was significantly associated with both IOP-Corvis as well as IOP-Pach in the FS-LASIK (β = 0.0261, *p* = 0.0003; β =  − 0.0451, *p* < 0.0001, respectively) and TPRK (β = 0.02746, *p* = 0.0021; β =  − 0.0435, *p* < 0.0001, respectively) groups. For mIOP, a less prominent relationship was observed in the TPRK group (β = 0.0057, *p* = 0.0216), and no significant association was found in the FS-LASIK group (β = 0.00646, *p* = 0.2503). For bIOP, no significant association was found in both groups (*p* = 0.7042 and 0.7407, respectively). However, age significantly affected bIOP in the TPRK group (β =  − 0.0523, *p* = 0.0228).

**Fig. 6:**
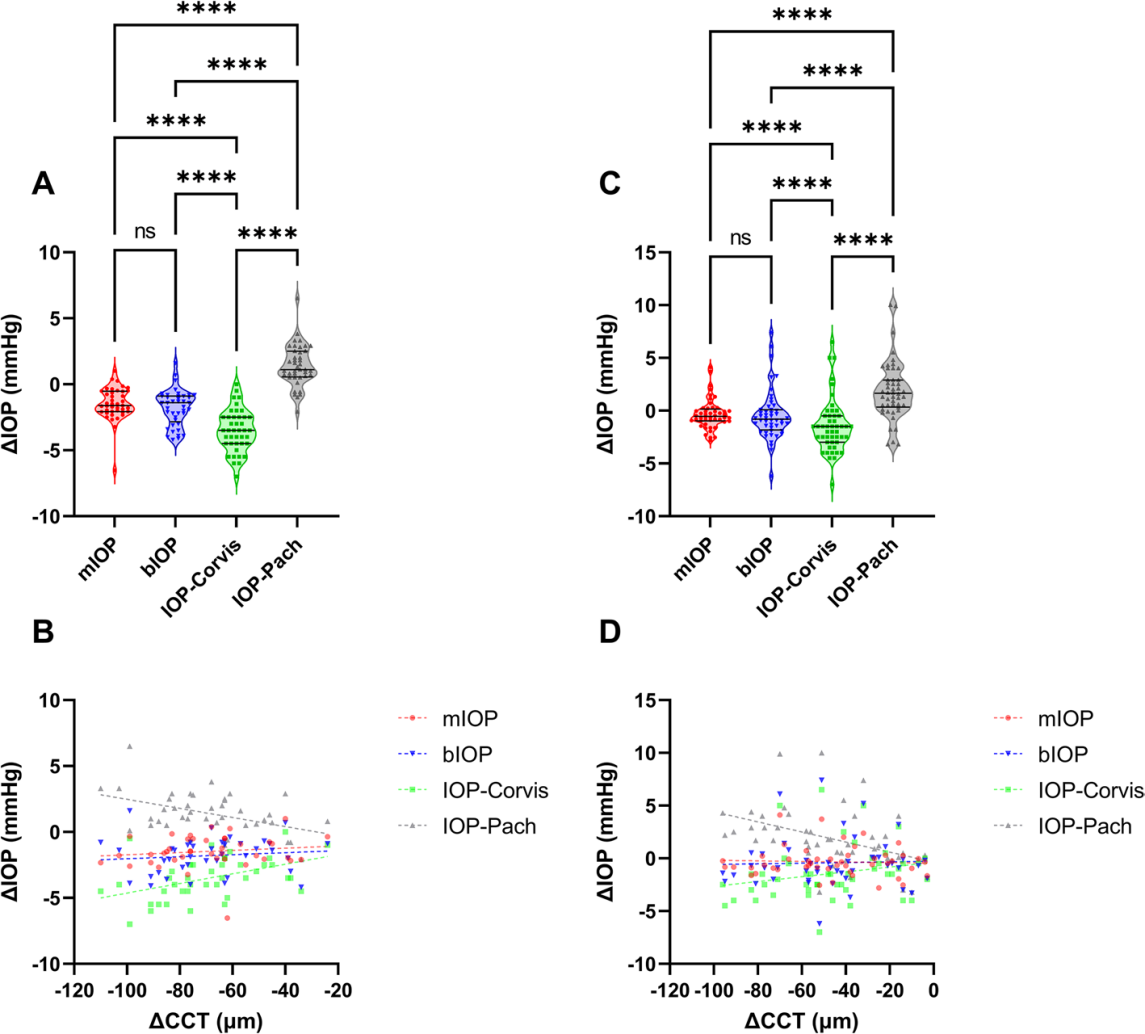
Distribution of postoperative IOP change and relationship with CCT change for four IOP methods (mIOP, bIOP, IOP-Corvis, and IOP-Pach) in the refractive surgery dataset. Results for FS-LASIK (*n* = 41) and TPRK (*n* = 48) patients are shown as violin plots in (**A**) and (**C**) respectively. Scatter plots illustrating the association between postoperative IOP and CCT changes in the FS-LASIK (**B**) and TPRK (**D**) patients are also shown. Pairwise comparisons in the violin plots are performed using repeated measures ANOVA (ns: not significant; *: *p* < 0.05; **: *p* < 0.01, ***: *p* < 0.001, ****: *p* < 0.0001)

Postoperatively, both bIOP and mIOP demonstrated no association with CCT, age, or R, regardless of the type of surgery received. IOP-Pach, on the other hand, remained strongly affected by CCT. Pairwise postoperative changes in IOP measurements were also compared using repeated measures ANOVA. In contrast with IOP-Corvis and IOP-Pach, which demonstrated a drastic reduction and elevation in postoperative values, respectively, the bIOP and mIOP values both remained nearly constant after surgery (Fig. 6A and B). Tukey’s multiple comparison further revealed no significant difference between ΔbIOP and ΔmIOP. Identical results were also obtained when plotting ΔIOP-Corvis, ΔIOP-Pach, ΔbIOP, and ΔmIOP against ΔCCT, with ΔbIOP and ΔmIOP demonstrating no significant association with ΔCCT. The horizontal line was better fitted in both cases (Fig. 6B and D).

**Fig. 7:**
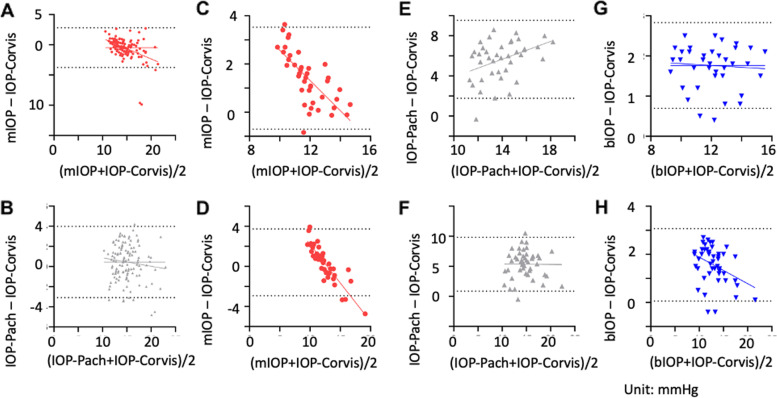
Bland–Altman plots showing agreement among four IOP methods (mIOP, bIOP, IOP-Corvis, and IOP-Pach). Results for the healthy validation (*n* = 129) (**A**, **B**), FS-LASIK (*n* = 41) (**C**, **E**, **G**), and TPRK (*n* = 48) (**D**, **F**, **H**) datasets are shown. Dashed lines represent the upper and lower limits of the 95% limits of agreement (LOA)

Next, Bland–Altman analysis was implemented to plot IOP-Corvis values against those of IOP-Pach, bIOP, and mIOP. Results for the healthy validation dataset (Dataset B) are displayed in Fig. 7A and B. The mean difference between mIOP and IOP-Corvis was − 0.47 mmHg; the 95% LOA was 6.56 mmHg, and both a strong fixed bias (*p* < 0.0001) and a weak proportional bias (*r*^2^ = 0.202; *p* < 0.0001) were present. IOP-Pach, however, demonstrated only a fixed bias (*p* = 0.0054) when compared with IOP-Corvis. The results for the FS-LASIK (Fig. 7C, E and G) and TPRK (Fig. 7D, F and H) groups consistently demonstrated a fixed bias and a strong proportional bias (*r*^2^ = 0.499/0.782, both *p* < 0.0001) between mIOP and IOP-Corvis values. However, no proportional bias was identified for bIOP in relation to IOP-Corvis in the FS-LASIK group (*p* = 0.6754), suggesting that the two methods only differ by a constant value, which contradicts the concept of bIOP as a modified measurement that adjusts for biomechanical properties.

### Validation in the in silico model

**Fig. 8:**
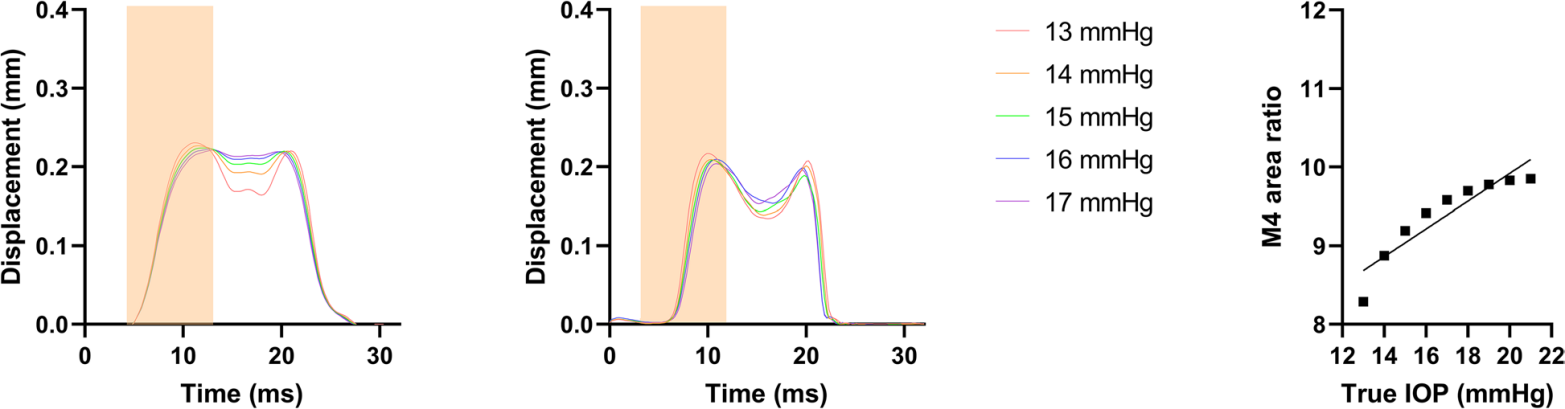
In silico-generated M_4_ modal profile and association between M_4_ ratio and true IOP based on the FEM model. The M_4_ modal profile of the FEM model and healthy participants of the same IOP are shown in (**A**) and (**B**), respectively. Shaded areas represent the rising phase. The linear regression results between M_4_ ratio and true IOP is shown in (**C**)

The validity of modal analysis was first analyzed by comparing the M_4_ modal shape between the finite element method (FEM) model of a given true intraocular pressure and the averaged results of patients with the same IOP (Fig. 8). For each IOP level, the modal curves were of similar shape, height, duration between the two groups, which also peaked and reached local minimum at roughly the same time, especially before and after the first applanation time. As intraocular pressure increases, modal curves in both groups exhibited earlier onset of the first peak and delayed onset of the second peaking, which resulted in a steeper uprising phase. Based on this good representability of real-world observations, the M_4_ ratio was then compared against true IOP in the in silico model, which revealed a significant high correlation between the two values under physiological settings (β = 0.1762, *r*^2^ = 0.8438, *p* = 0.0005).

## Discussion

High-speed Scheimpflug images of the right eyes in a Taiwanese population were analyzed in this study. In this proof-of-concept study, we sought to compare the performance of mIOP (a physically-interpretable index that reflects corneal vibration) with a well-established and widely-applied IOP method. Although GAT is considered the gold standard for IOP measurement, its usage has decreased since the COVID-19 pandemic due to the risk of virus spread. Fortunately, the data obtained from a noncontact tonometer shows that there exists a correlation between Covis-IOP and GAT [4, 18, 29], which could help to bridge the gap between mIOP and GAT. Given that GAT-derived IOP is lacking in the population sample used in this study, we plan to conduct more GAT measurements in the future to enable a more detailed comparison with mIOP.

We proposed a Legendre polynomial basis for detailed interrogation of corneal biomechanical properties and IOP measurement (mIOP) through decoupling cornea deformation profiles into orthogonal modes. Legendre decomposition has been widely applied in the fields of engineering; this study is the first to apply Legendre polynomials to corneal vibration. Similar studies suggest the use of Chebyshev polynomials to the curves of the corneal deformation [23] or an intuitive point-to-point comparison [30]. However, our method supports a wide range of modes including even/odd modes, and is built on the orthogonal modalities. The advantage of this method is that in the deformation profiles, temporal changes in each modal coefficient leads to subtle changes in corneal asphericity, which can easily be quantified.

The breathing mode_,_ high-order even modes, and odd modes were all excluded from our model, as they respectively reflect whole eye movement, over-undulated curves incompatible with real-world deformation, and regional weakening associated with certain cornea ectasia conditions. The mIOP is thus based on a single mode, M_4_, in which the sequence of events from the first air puff of air making contact to the first applanation characterizes the shifting equilibrium between external (air puff) and internal (IOP) forces. Whereas conventional IOP models are derived from analyzing a single timepoint (i.e., the first applanation), mIOP offers a novel opportunity to discuss intraocular pressure in a dynamic manner. Additionally, this modal approach effectively functions as a “noise” filter, removing the factors of pathological, rotational, and misaligned movement, which could interfere with IOP measurement. In combination with its self-calibration property as a ratio-based index, the mIOP further minimizes interpatient variability due to measurement error.

The validity of mIOP was verified both on the population and biomechanical levels. The former includes healthy subjects and those who underwent refractive surgery: as with bIOP, the strength and degree of mIOP’s association with age, R, and CCT were mostly weak and negligible in both groups. We further found that the performance of mIOP was comparable with that of bIOP with respect to the magnitude of change in postoperative IOP in the refractive surgery dataset, suggesting that the two methods produce relatively conserved pre- and postoperative measurements regardless of surgery type or degree of CCT thinning. From the biomechanical aspect, the M_4_ ratio correlated well with true intraocular pressure under physiological levels in the FEM model, again indicating modal analysis as an efficient method for separation of pressure-related signals from material properties. In contrast to the complicated 19 coefficient–based bIOP formula generated through complex statistical approaches, the simplicity of mIOP appears much more physically self-explanatory and straightforward. Taken together, this study demonstrated mIOP to be a reliable and intuitive method for IOP measurement independent of corneal material or morphological properties. However, this study has some limitations. Firstly, GAT data were unavailable. Secondly, non-Asian populations or patients with common ocular diseases, including glaucoma, were not included. Thirdly, the temporal rhythm of IOP, which could play a role in intra- and inter-patient IOP variations, was not taken into account due to its complex nature and the lack of well-established circadian IOP models [31]. The repeatability and stability of the tonometer is also determined in this study [32].

Further studies are warranted to examine the applicability of mIOP under pathological conditions and non-physiological IOP levels. Specifically, ex vivo validations, either based on suitable animal models or cadaveric eyes, would be crucial in establishing the independence of mIOP from CCT, age, and other corneal biomechanical parameters.

However, the primary aim of this study was to explore the effectiveness of modal analysis as a novel IOP measurement that is biomechanically meaningful in routine practice. Taken together with the results of FEM simulations, mIOP has demonstrated good reliability and reproducibility as a purely biomechanical based IOP approach that does not rely on input other than the deformation curve itself.

## Conclusions

In summary, the mIOP is generated from the rising phase of M_4_, such that deformation of the central one-fifth portion of the cornea is emphasized while irrelevant vibrations and deviations are minimized, thereby providing an accurate account of force dynamics during air-puff contact. The performance of mIOP in both general ophthalmology and refractive surgery is comparable to that of existing IOP measurement methods. To our knowledge, this is the first study of its kind to apply classic orthogonal polynomials to corneal profiles to measure IOP. The study thus introduces a new energy-based perspective for understanding IOP that may supplement current single-timepoint macroscopic characterization or parameterized modeling.

## Supplementary Information

**Additional file 1.** Illustrationof image processing workflow. 

**Additional file 2.** Listof input parameters and values of the *in silico* model.

**Additional file 3.** Definitions of all 65 primary modal analysis–derived parameters. 

**Additional file 4.** Univariate linear regression of the four IOP methods (mIOP,IOP-Corvis, IOP-Pach, and bIOP) with age, R, CCT, and A1T in the refractivesurgery dataset. Preoperativeand postoperative results for FS-LASIK and TPRK patients (n = 41 and 48 respectively)are shown. 

## Data Availability

The datasets used and/or analyzed during the current study are available from the corresponding author on reasonable request.

## Abbreviations

A1T: Time to the first applanation
ANOVA: Analysis of variance
bIOP: Biomechanically corrected intraocular pressure
CCT: Central corneal thickness
FEM: Finite element method
FS-LASIK: Femtosecond-assisted laser in situ keratomileusis
GAT: Goldmann applanation tonometer
IOP: Intraocular pressure
IOP-Corvis: Corvis ST-derived intraocular pressure
IOP-Pach: Pachymetry-adjusted intraocular pressure
LOA: Limits of agreement
mIOP: Modal analysis-derived intraocular pressure
R: Corneal curvature radius
TPRK: Transepithelial photorefractive keratectomy
WEM: Whole eye movement
