# Supplementary figures and images for: Development and validation of an intuitive biomechanics-based method for intraocular pressure measurement: a modal analysis approach
Source: BMC Ophthalmol. 2023 Mar 27;23:124. doi: 10.1186/s12886-023-02867-8 (PMC10041475; doi:10.1186/s12886-023-02867-8)

**Additional file 1 : Illustration of image processing workflow.**

**
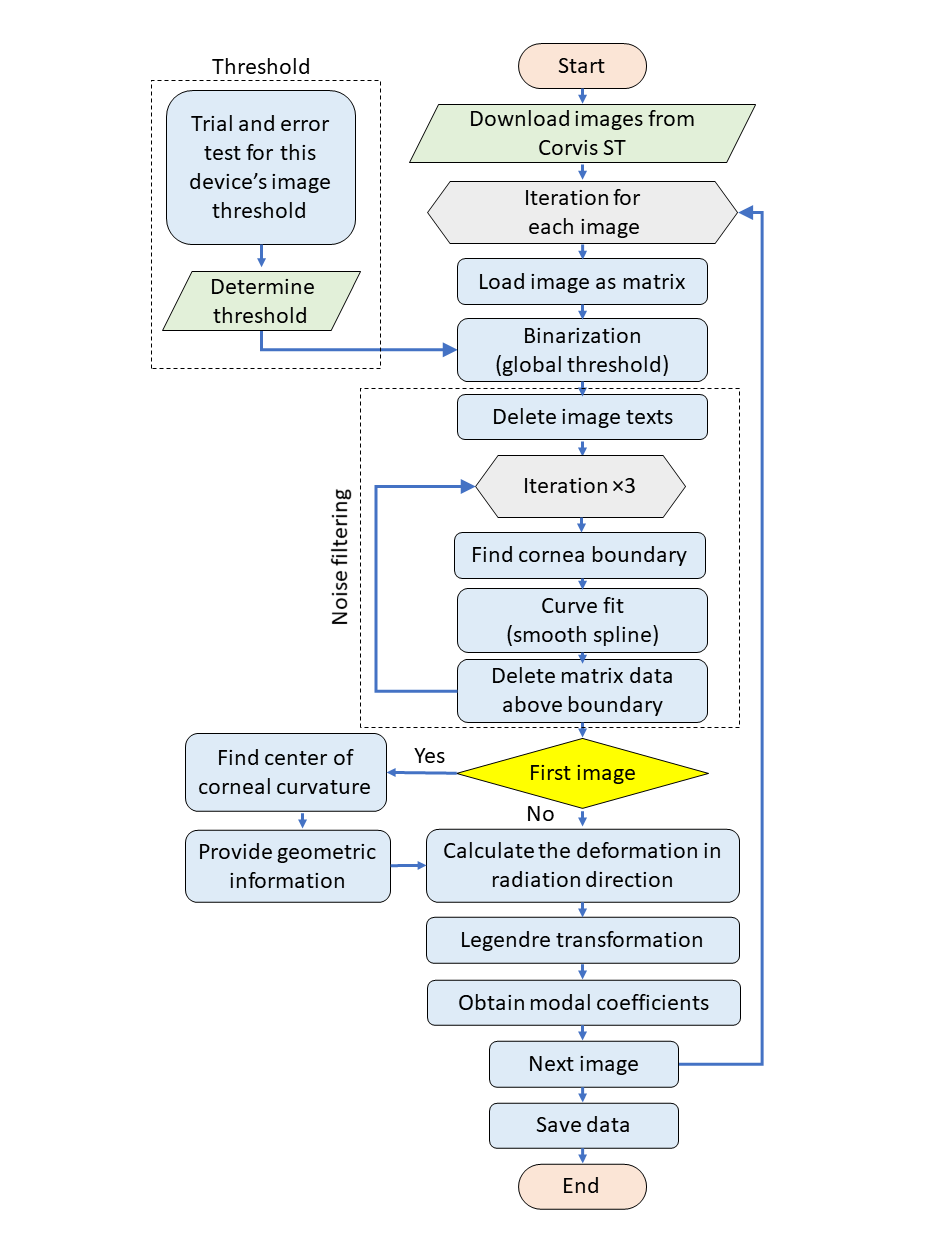
**

Supplement: Supplementary file 1 — Additional file 1. Illustrationof image processing workflow. [file 12886_2023_2867_MOESM1_ESM.docx]
