# Supplementary material for: Development and validation of an intuitive biomechanics-based method for intraocular pressure measurement: a modal analysis approach
Source: BMC Ophthalmol. 2023 Mar 27;23:124. doi: 10.1186/s12886-023-02867-8 (PMC10041475; doi:10.1186/s12886-023-02867-8)
Supplement: Supplementary file 2 — Additional file 2. Listof input parameters and values of the in silico model. [file 12886_2023_2867_MOESM2_ESM.docx]

**Additional file 2 : List of input parameters and values of the *in silico* model.**

| **Geometrical parameters** | **Value** |
| --- | --- |
| Vertical diameter [mm] | 11 |
| Horizontal diameter [mm] | 11 |
| Anterior surface radius [mm] | 10.5 |
| Posterior surface radius [mm] | 9.4 |
| Central corneal thickness [µm] | 500 |
| Limbal corneal thickness [µm] | 1000 |
| Air puff nozzle radius [mm] | 1.8 |
| Air puff nozzle-corneal apex distance [mm] | 11 |
|  |  |
| **Ogden hyper-elastic parameters** | **Value** |
| μ [mPa] | 0.0486 |
| α [mm] | 60 |
| D (Compressibility) [mm] | 0.00000001 |
| **Maxwell viscio-elastic parameters** | **Value** |
| Energy factor | 0.3 |
| Relaxation time constant [s] | 0.001 |
| **Other material parameters** | **Value** |
| Density [kg/m^3^] | 1844 |
| Poisson’s ratio | 0.49 |
| Young’s modulus [mPa] | 0.22 |
|  |  |
| **Air puff field parameters** | **Value** |
| Vmax [m/s] | 125 |
| α1 | -0.8 |
| α2 | -2 |
| s1_Max_ | 10 |
| s1_Min_ | -7 |
| s2_Max_ | 5 |
| s2_Min_ | -14 |

where $V=V_{max}{(1+e^{-S_{n}})}^{\alpha_{n}}$,

$S_{1}=\frac{t}{0.0145}\left( S_{1\mathrm{Max}}-S_{1\mathrm{Min}} \right)+S_{1Min}$ ,

$S_{2}=S_{2Max}-\frac{t-0.0145}{0.0145}\left( S_{2\mathrm{Max}}-S_{2\mathrm{Min}} \right)$ , $n=\left\{ \begin{aligned} 1 \mathrm{if} t\leq0.0145 \\ 2 \mathrm{if} t>0.0145 \end{aligned} \right.$ .
