# Supplementary material for: Development and validation of an intuitive biomechanics-based method for intraocular pressure measurement: a modal analysis approach
Source: BMC Ophthalmol. 2023 Mar 27;23:124. doi: 10.1186/s12886-023-02867-8 (PMC10041475; doi:10.1186/s12886-023-02867-8)
Supplement: Supplementary file 3 — Additional file 3. Definitions of all 65 primary modal analysis–derived parameters. [file 12886_2023_2867_MOESM3_ESM.docx]

**AdAdditional file 3 : Definitions of all 65 primary modal analysis–derived parameters.**

**
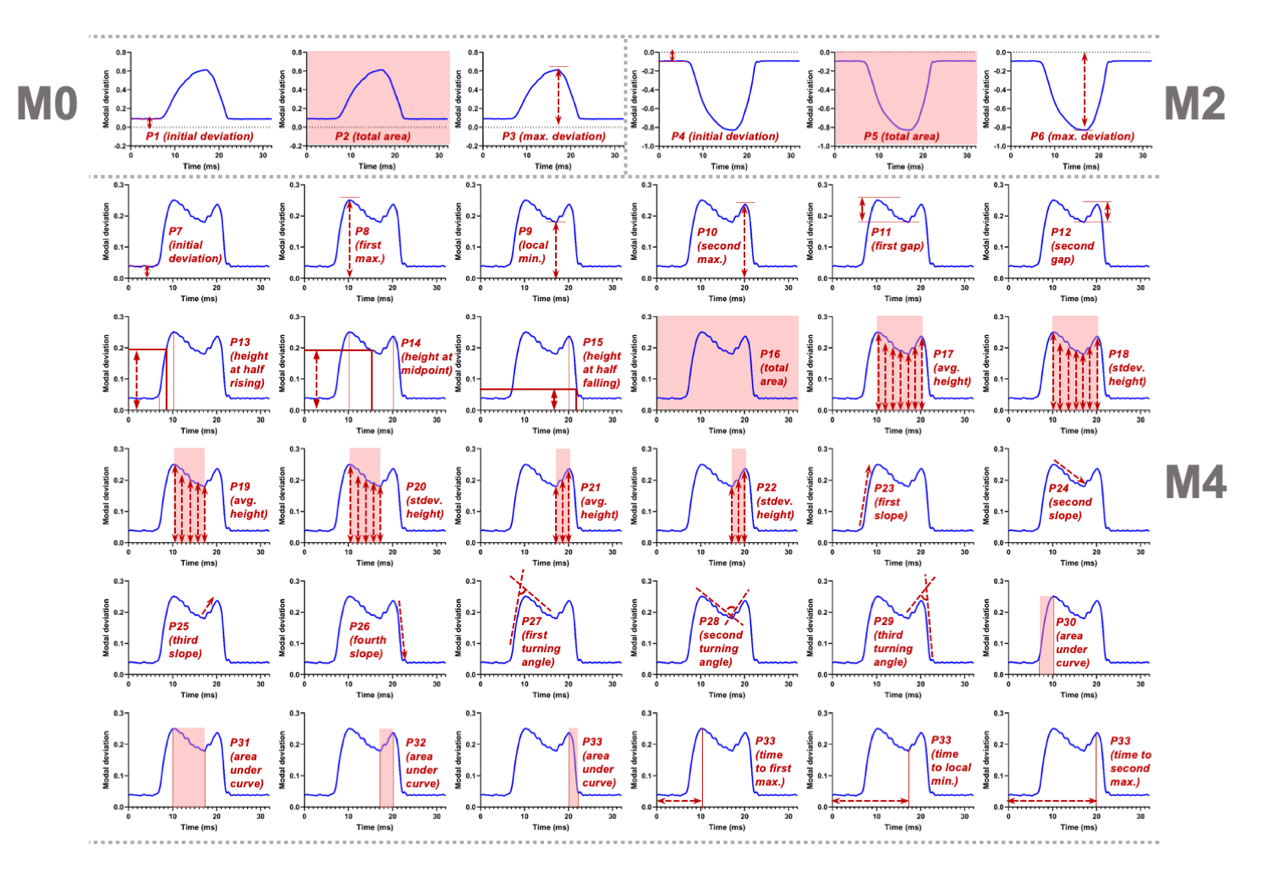
**

**
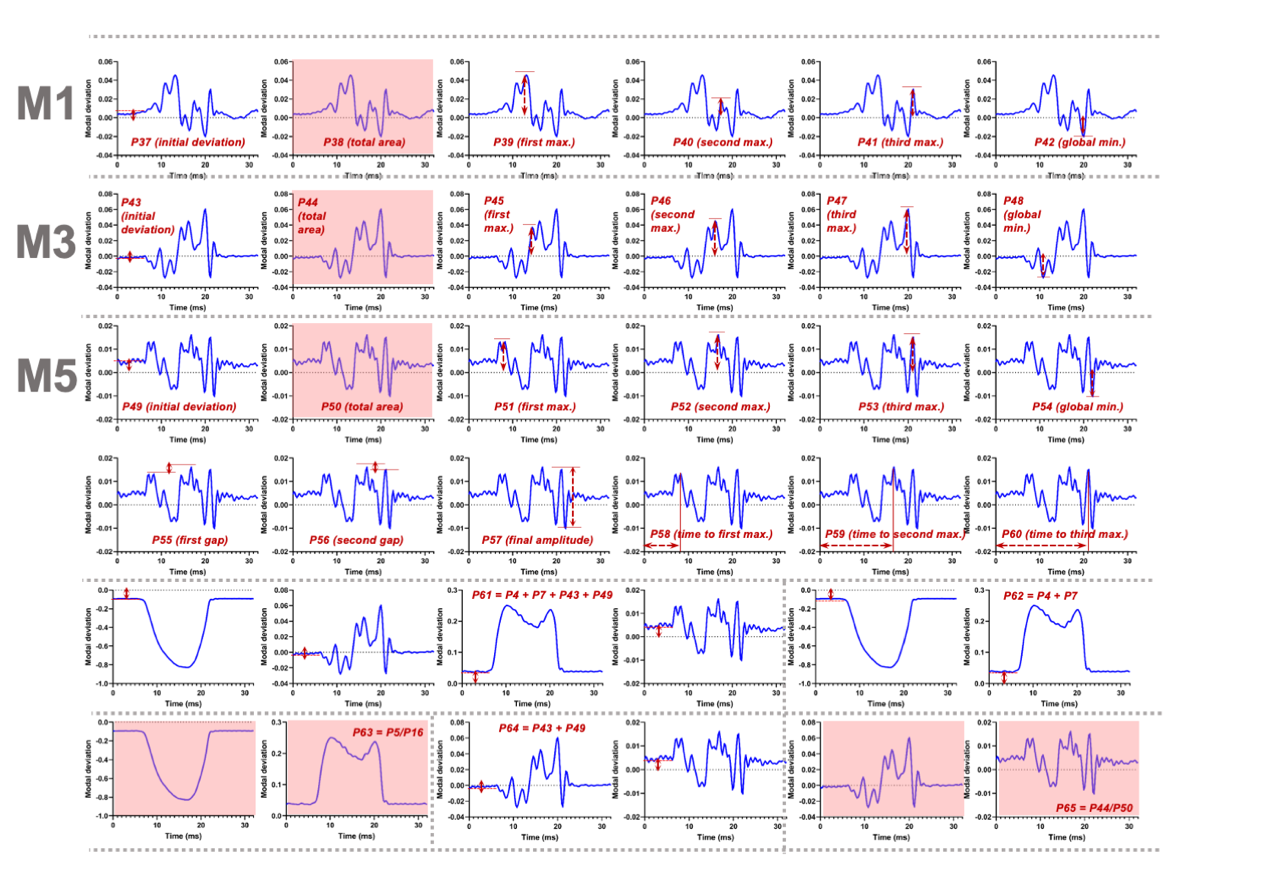
**

**
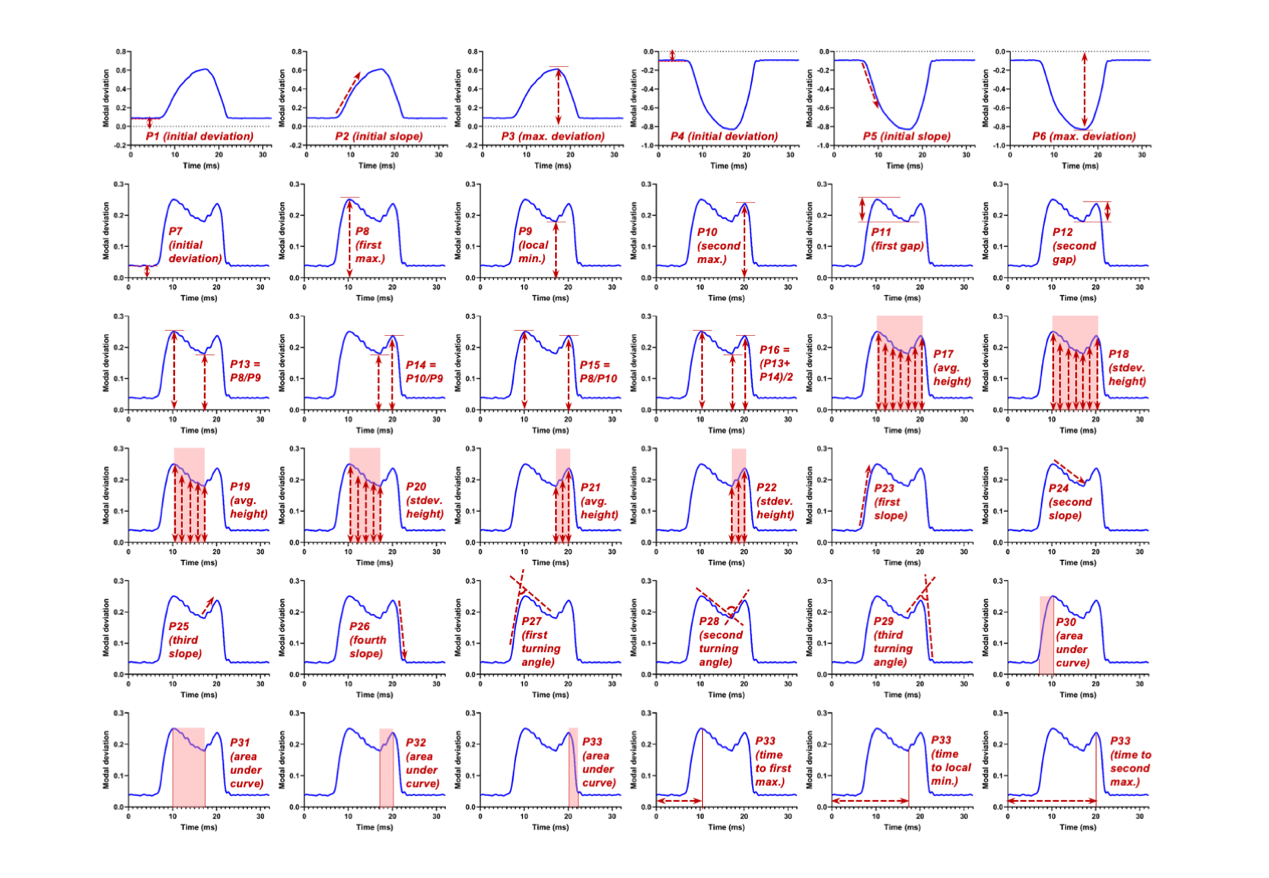
**

**ditional file 3 : Definitions of all 65 primary modal analysis–derived parameters.**

**
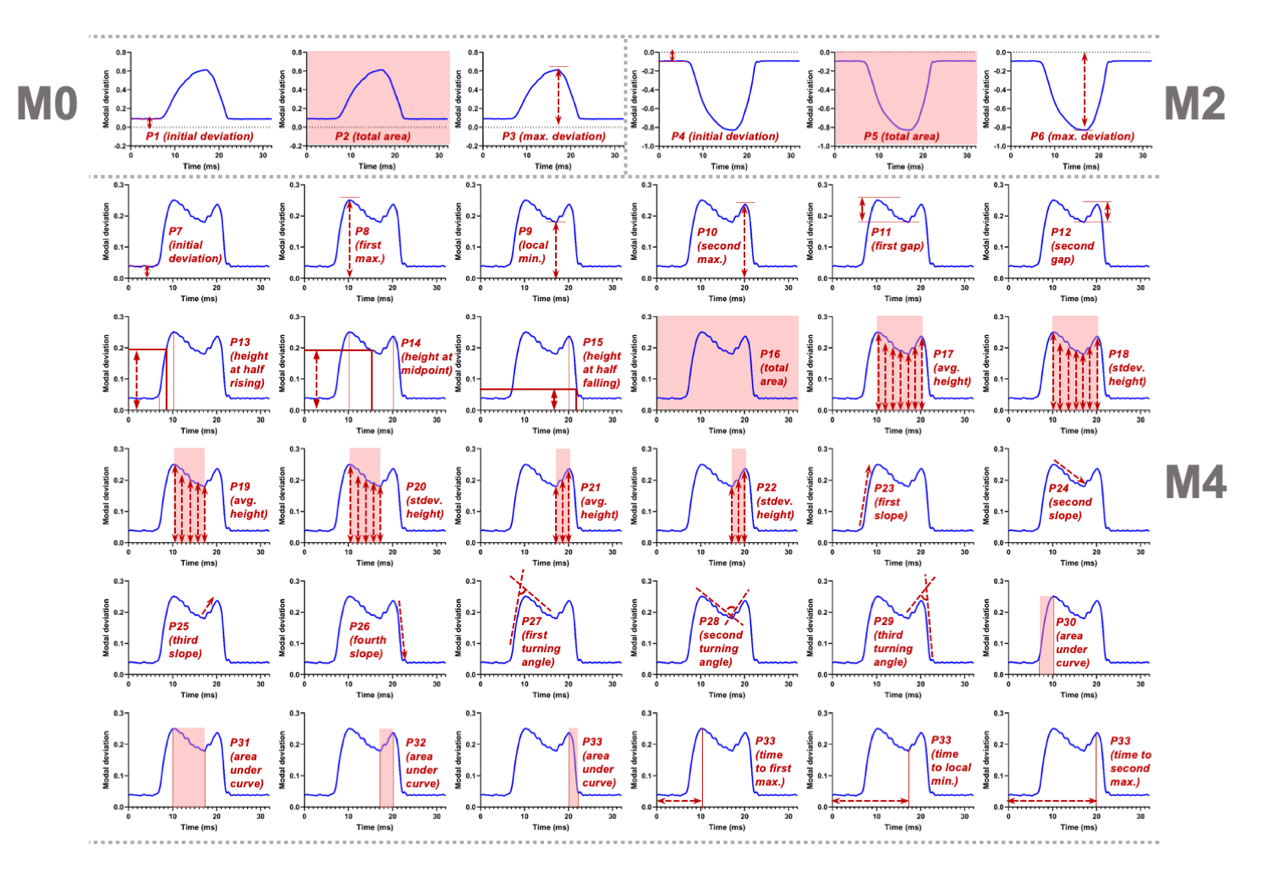
**

**
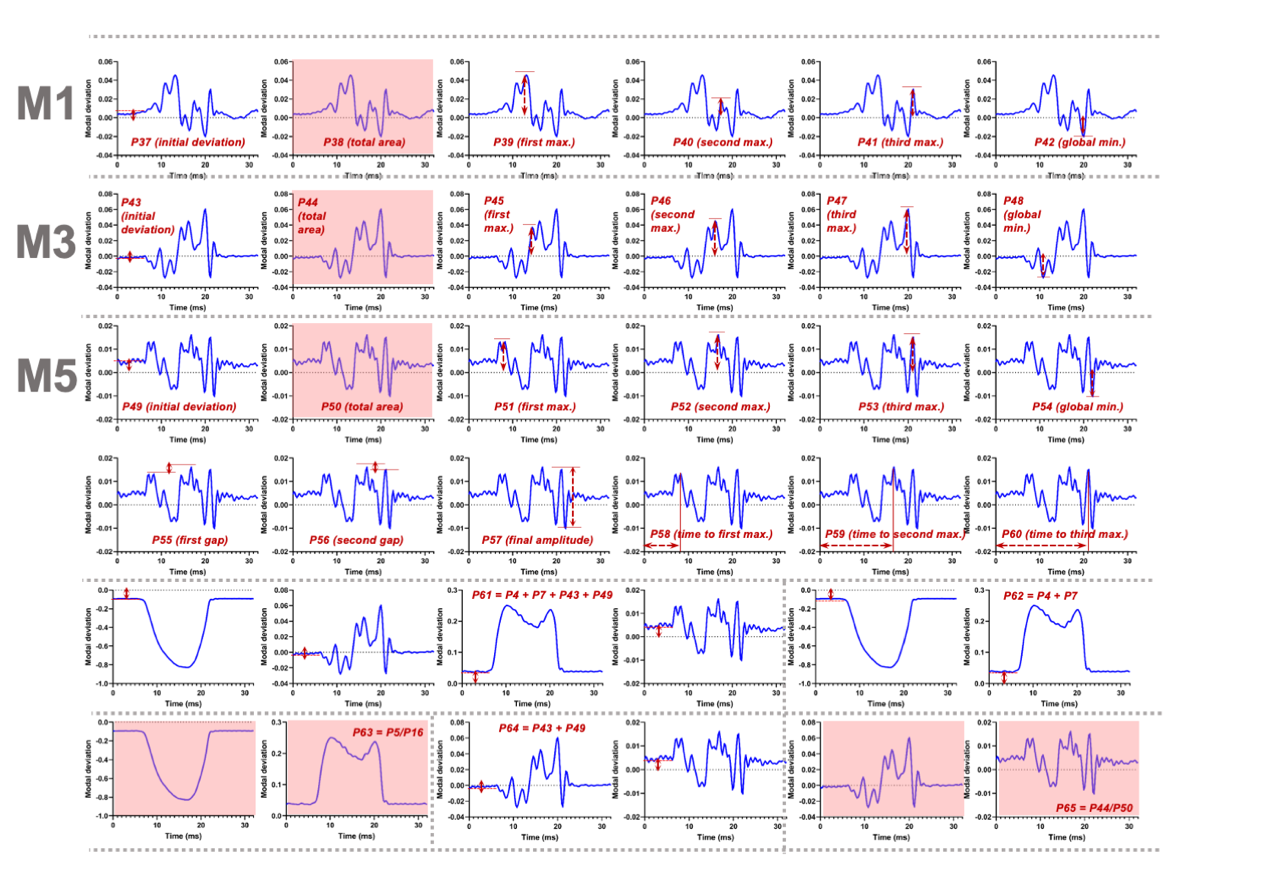
**

**
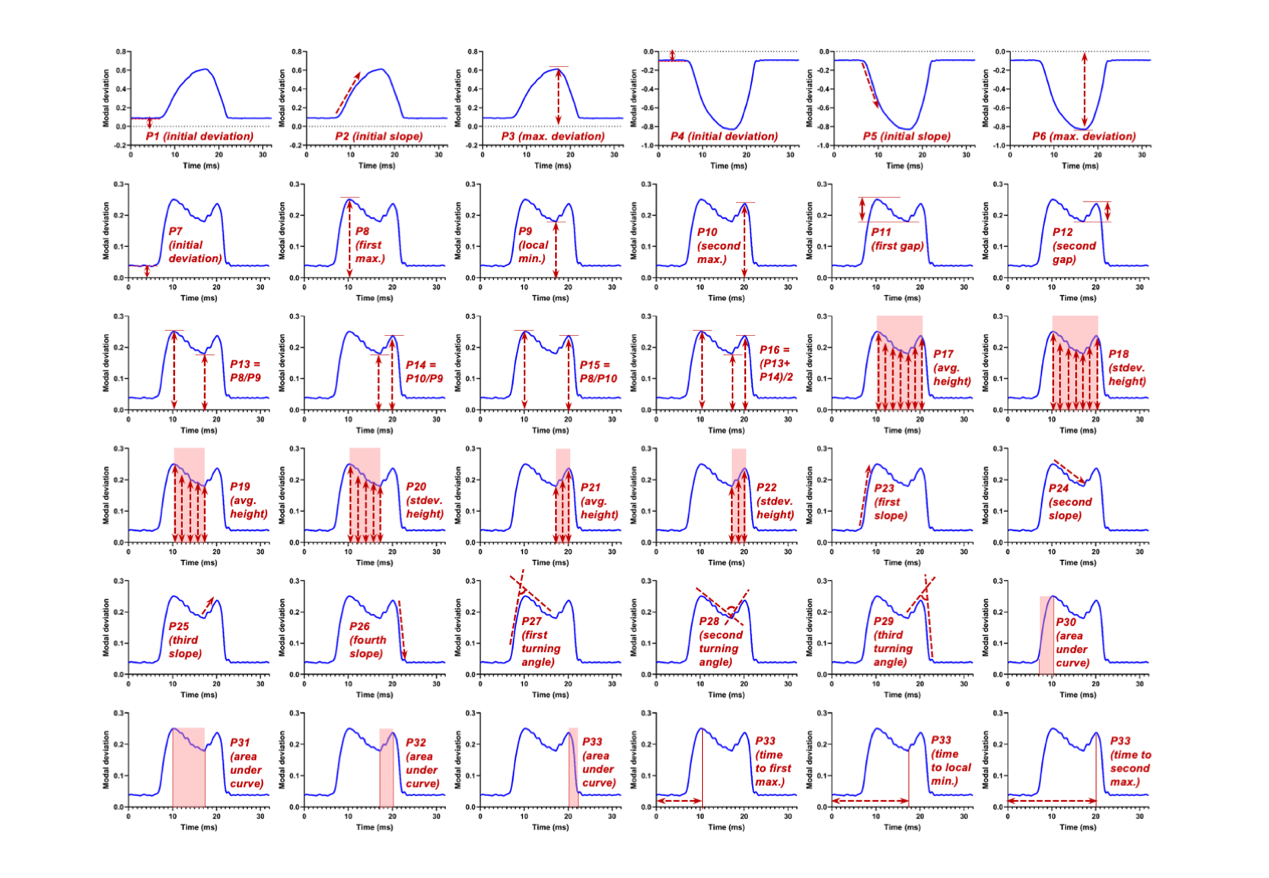
**
