# Supplementary material for: Development and validation of an intuitive biomechanics-based method for intraocular pressure measurement: a modal analysis approach
Source: BMC Ophthalmol. 2023 Mar 27;23:124. doi: 10.1186/s12886-023-02867-8 (PMC10041475; doi:10.1186/s12886-023-02867-8)
Supplement: Supplementary file 4 — Additional file 4. Univariate linear regression of the four IOP methods (mIOP,IOP-Corvis, IOP-Pach, and bIOP) with age, R, CCT, and A1T in the refractivesurgery dataset. Preoperativeand postoperative results for FS-LASIK and TPRK patients (n = 41 and 48 respectively)are shown. [file 12886_2023_2867_MOESM4_ESM.docx]

**Additional file 4 : Univariate linear regression of the four IOP methods (mIOP, IOP-Corvis, IOP-Pach, and bIOP) with age, R, CCT, and A1T in the refractive surgery dataset.** Preoperative and postoperative results for FS-LASIK and TPRK patients (n = 41 and 48 respectively) are shown.

| **Table 5** | | **Pre-op** | | **Post-op** | | **Pre-op TPRK** | | **Post-op TPRK** | | |
| --- | --- | --- | --- | --- | --- | --- | --- | --- | --- | --- |
|  |  | **FS-LASIK** | | **FS-LASIK** | |  |  |  |  |  |
|  |  | **β** | **p value** | **β** | **p value** | **β** | **p value** | **β** | **p value** |  |
| **mIOP** | **Age (yrs)** | 0.005491 | 0.8168 | 0.008843 | 0.5812 | -0.01017 | 0.5604 | -0.01544 | 0.4483 |  |
|  | **R (mm)** | 0.03067 | 0.895 | -0.1137 | 0.6966 | -0.1079 | 0.7006 | -0.1855 | 0.5237 |  |
|  | **CCT (µm)** | 0.006462 | 0.2503 | 0.005215 | 0.3214 | 0.005697 | 0.0216 | 0.005571 | 0.0568 |  |
|  | **A1T (ms)** | 4.563 | <0.0001 | 3.757 | <0.0001 | 4.759 | <0.0001 | 3.464 | <0.0001 |  |
| **IOP-Corvis** | **Age (yrs)** | 0.006888 | 0.8069 | 0.005566 | 0.8463 | -0.03694 | 0.1688 | -0.03015 | 0.4929 |  |
|  | **R (mm)** | 0.4436 | 0.1025 | 0.6711 | 0.1937 | 0.5162 | 0.2319 | 0.8074 | 0.1952 |  |
|  | **CCT (µm)** | 0.0261 | 0.0003 | 0.01061 | 0.2606 | 0.02746 | 0.0021 | 0.03647 | 0.0023 |  |
|  | **A1T (ms)** | 6.34 | <0.0001 | 6.631 | <0.0001 | 7.394 | <0.0001 | 7.702 | <0.0001 |  |
| **IOP-Pach** | **Age (yrs)** | 0.02289 | 0.5175 | 0.01916 | 0.6378 | -0.01442 | 0.6373 | -0.08143 | 0.0567 |  |
|  | **R (mm)** | -0.4285 | 0.2131 | -0.2328 | 0.7532 | -0.5422 | 0.2666 | -0.6956 | 0.2603 |  |
|  | **CCT (µm)** | -0.04508 | <0.0001 | -0.06051 | <0.0001 | -0.04346 | <0.0001 | -0.03448 | 0.0037 |  |
|  | **A1T (ms)** | 1.217 | 0.3817 | 7.126 | <0.0001 | 3.612 | 0.0058 | 5.102 | <0.0001 |  |
| **bIOP** | **Age (yrs)** | -0.0097 | 0.6532 | -0.0101 | 0.719 | -0.05231 | 0.0228 | -0.06296 | 0.1064 |  |
|  | **R (mm)** | 0.0868 | 0.6816 | 0.4467 | 0.3793 | 0.06431 | 0.8654 | 0.3459 | 0.6186 |  |
|  | **CCT (µm)** | -0.00229 | 0.7042 | -0.00717 | 0.439 | 0.002712 | 0.7407 | 0.01423 | 0.2035 |  |
|  | **A1T (ms)** | 3.762 | <0.0001 | 6.544 | <0.0001 | 5.811 | <0.0001 | 6.681 | <0.0001 |  |
